# Case report: Candidiasis of gastrojejunostomosis after pancreaticoduodenectomy: Preliminary experience from two cases

**DOI:** 10.3389/fonc.2022.888927

**Published:** 2022-08-24

**Authors:** Thanh Khiem Nguyen, Ham Hoi Nguyen, Cong Long Nguyen, Tuan Hiep Luong, Long Doan Dinh, Van Duy Le, Kim Khue Dang, Thi Lan Tran

**Affiliations:** ^1^ Department of Gastrointestinal and Hepato-Pancreato-Biliary Surgery, Bach Mai Hospital, Hanoi, Vietnam; ^2^ Department of Gastroenterology and Hepatology, Bach Mai Hospital, Hanoi, Vietnam; ^3^ University of Medicine and Pharmacy, Vietnam National University, Hanoi, Vietnam; ^4^ Department of Surgery, Hanoi Medical University, Hanoi, Vietnam; ^5^ Pathology Center, Bach Mai Hospital, Hanoi, Vietnam

**Keywords:** candidiasis, pancreaticoduodenectomy, case report, pancreactic cancer, gastrojejunostomosis

## Abstract

**Introduction:**

Invasive *Candida* infection, or candidiasis, especially in gastrointestinal tract (GIT) is an infrequent but aggressive disease caused by *Candida* species. Candidiasis of gastrojejunostomosis after extensive gastrointestinal surgery may cause serious complications such as perforative peritonitis and anastomotic stenosis, which requires surgical interventions.

**Case presentation:**

Our two patients had undergone pancreaticoduodenectomy (PD), respectively, due to pancreatic ductal adenocarcinoma and intraductal papillary mucinous neoplasms of the pancreatic head. Both the patients were malnutritioned and debilitated before the surgery, and they required reoperation for postoperative Candidiasis-relevant complication.In the first case, the patient was readmitted to the hospital with symptoms of perforative peritonitis, for which he underwent surgery and had *Candida* found in both gastrojejunostomosis ulcer and peritoneal fluid. In our second case, the patient was admitted to the hospital twice after the first operation and diagnosed with *Candida*-induced gastrojejunostomosis stenosis by esophagogastroduodenoscopy (EGD) and endoscopic biopsy. Fluconazole was indicated for a 2-week regimen. Blood sample withdrawn afterward showed no evidence of fungal agents, and the anastomotic stenosis responded well to treatment. However, after 3 weeks, he came back with cachexia and symptoms of gastrojejunostomotic stenosis. EGD showed no image of fungal agents but anastomotic stenosis due to chronic inflammatory process. The patient was then reoperated to redo his gastrojejunostomosis.

**Conclusion:**

Candidiasis of gastrojejunostomosis after extensive gastrointestinal surgery such as PD is a very aggressive condition that may cause perforative peritonitis and anastomotic stenosis. However, there have been no publications on this disorder, and the strategic treatment remains unknown. We hereby present a report of two cases with postoperative gastrojejunostomosis candidiasis presenting with non-specific but aggressive and early clinical symptoms.

## Introduction

Invasive *Candida* infection, or candidiasis, especially in gastrointestinal tract (GIT) is an infrequent but aggressive disease caused by *Candida* spp. (1). These *Candida* species can be detected in the healthy human GIT, with incidence ranging from 30% to 60% (2). However, in immunocompromised or/and cachectic patients, or patients with injudicious use of antibacterial agents, or immunocompetent patients with prolonged use of antacid drugs, *Candida* spp. may cause opportunistic infection (3). There are at least 15 pathogenic *Candida* species, and over 90% cases were reported to be caused by *Candida albicans*, *Candida tropicalis*, *Candida parapsilosis*, *Candida glabrata*, and *Candida krusei* (1).

Gastrojejunostomosis candidiasis after extensive gastrointestinal surgery like pancreaticoduodenectomy (PD) is a very aggressive condition that may cause serious complications such as perforative peritonitis and anastomotic stenosis, which requires surgical interventions. However, there have been no publications on this disorder, and the strategic treatment remains unknown. Herein, we report two cases with gastrojejunostomosis candidiasis after PD, leading to anastomotic stricture in one case and perforation in the other, and present our preliminary experience for these complications. All our work has been reported in line with the SCARE criteria and guidelines (4).

## Case presentation

### Case 1

**Figure 1:**
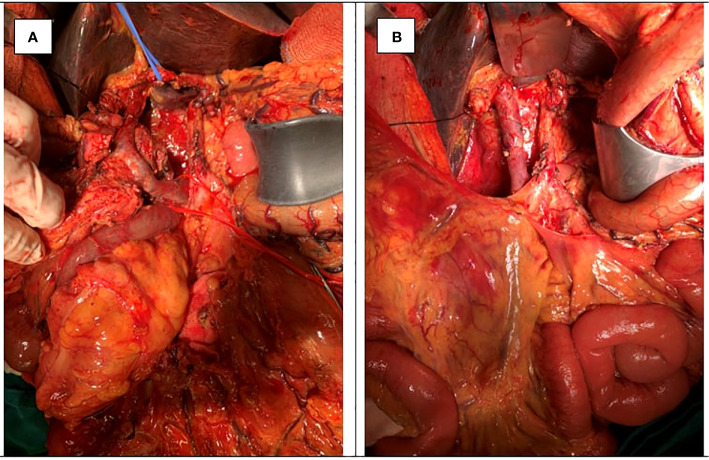
A 73-year-old man with pancreatic ductal adenocarcinoma (PDAC) with PV involvement (pT4N2M0 or Stage III according to AJCC Staging 2017) **(A)** underwent pancreaticoduodenectomy (PD) and total pancreatectomy with extended lymphadenectomy and segmental portal vein (PV) resection **(B)**.

A 73-year-old man underwent PD and total pancreatectomy with extended lymphadenectomy and segmental portal vein (PV) resection for pancreatic ductal adenocarcinoma (PDAC) with PV involvement (pT4N2M0 or Stage III according to AJCC Staging 2017) (**Figure 1**) (5). The patient had used of prolonged antibacterial (Cephalosporin third generation and Metronidazole for prophylaxis) and antacid drugs as well as total parenteral nutrition postoperatively. On postoperative day (POD) 4, the patient underwent reoperation due to bleeding from right marginal colic artery. On POD 10, after starting refeeding, over 1,000 ml of slightly milky ascites was discharged from the inserted drain. A low-fat, middle-chain triglyceride (MCT) diet was prescribed, but there were no improvements in 10 PODs. On POD 20, intranodal lymphangiography was consulted and performed, and approximately 8 ml of mixture of Aetoxisclerol and air in 1:3 ratio was injected around the right and left PVs. After the intervention, MCT diet was prescribed, and drainage decreased to 700 ml on the following day and to approximately 300 ml of serous fluid on the following seventh day. The drain output remained low, and after 10 days, the drainage was 50 ml, and all drains were removed.

**Figure 2:**
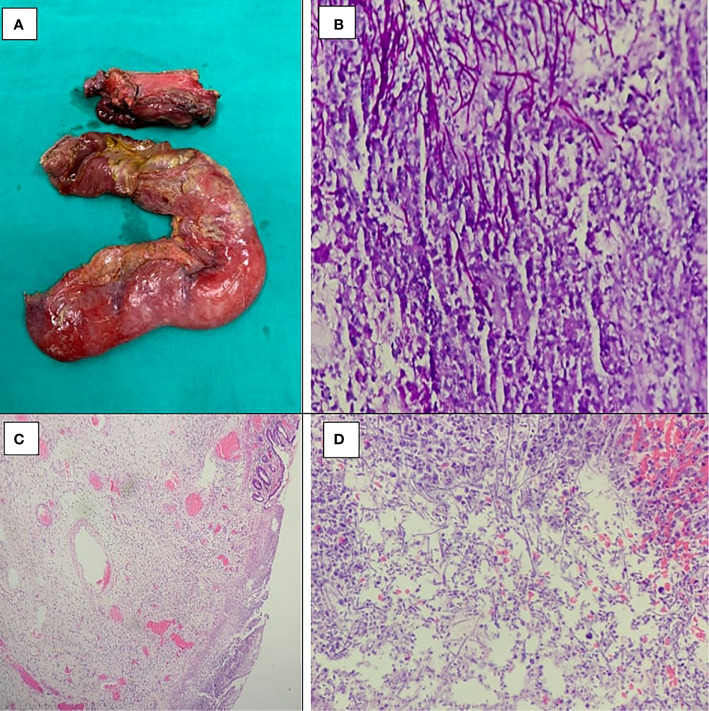
**(A)** The gastrojejunostomosis perforation with peritoneal fluid. PASx400 **(B)**, HEx40 **(C)**, and HEx400 **(D)** showed *Candida* spores and budding hyphae invading and destroying adjacent gastric wall and surrounding non-specific inflammation.

However, 1 month after the first discharge, the patient was readmitted to the hospital with an aggressive syndrome of septic shock. His abdomen was rigid with generalized guarding; abdominal x-ray showed subdiaphragmatic free gas. Diagnosed with gastrojejunostomosis perforation, he underwent an urgent laparotomy. The intraoperative diagnosis was septic shock/peritonitis due to perforated gastrojejunal anastomotic ulcers. A wedge biopsy from the gastrojejunostomosis ulcer margin and peritoneal fluid was taken, and *Candida* was found in both samples (**Figure 2**). Subtotal gastrectomy and jejunectomy with gastrojejunostomosis redo surgery was performed. Fluconazole was indicated for 2 weeks, and blood sample was rechecked to find no evidence of fungal agents left. The patient had postoperative gastrojejunostomosis leakage but no peritonitis. However, the patient was too ill for a reoperation and, therefore, was discharged while waiting for further interventions. After 6 months, hepatic and peritoneal metastasis were detected. The patient and his family decided to refuse any further interventions.

### Case 2

**Figure 3:**
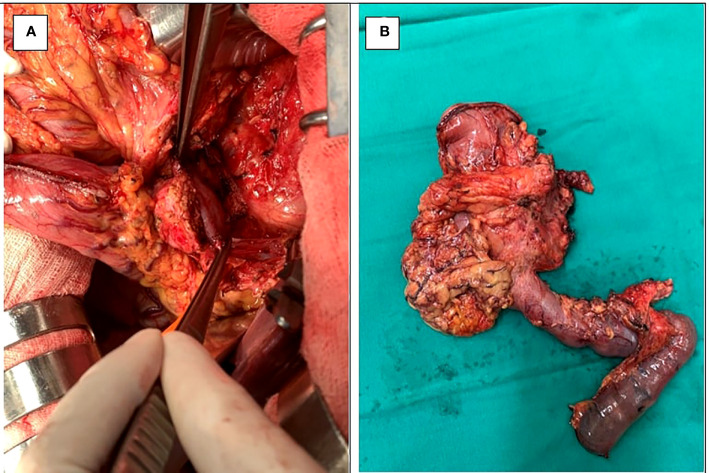
A 65-year-old man with intraductal papillary mucinous neoplasms (IPMN) of the pancreatic head **(B)** underwent pancreaticoduodenectomy (PD) with extended lymphadenectomy **(A)**.

A 65-year-old man underwent PD with extended lymphadenectomy for intraductal papillary mucinous neoplasms (IPMN) of the pancreatic head (**Figure 3**). The patient had used of prolonged antibacterial (Cephalosporin third generation and Metronidazole for prophylaxis) and antacid drugs as well as total parenteral nutrition postoperatively. In POD 7, The patient had symptoms of anastomotic stenosis, including continuous nausea and vomiting. The patient had to undergo a reconstructive surgery to expand the jejunum and stomach and dilate the gastrojejunostomosis. The patient responded well to treatment and was discharged on POD 20.

**Figure 4:**
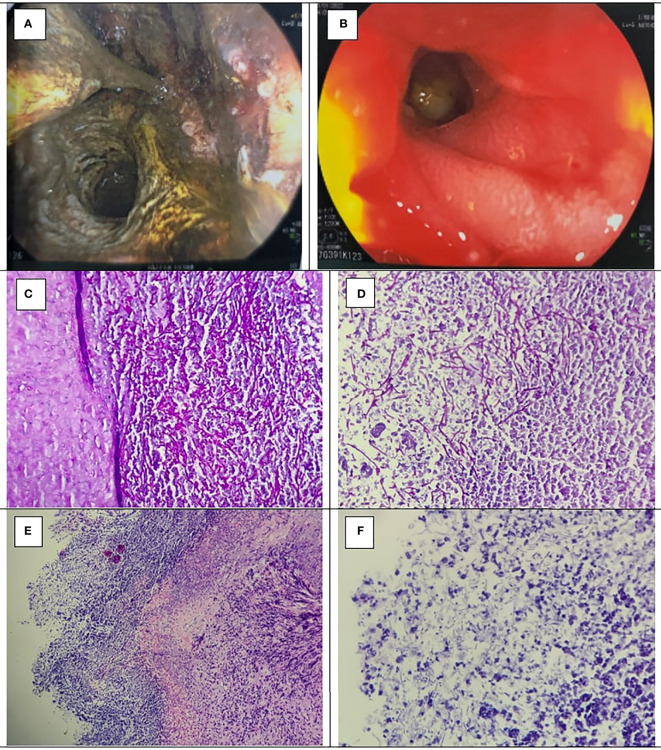
**(A)** Gastrojejunostomosis stenosis confirmed by gastrointestinal endoscopy. **(B)** The gastrojejunostomosis was good response after 2 weeks of Fluconazole prophylaxis. PASx400 **(C, D)**, HEx100 **(E)**, and Hex400 **(F)** showed *Candida* spores and budding hyphae invading and destroying adjacent gastric wall, and surrounding non-specific inflammation.

However, the patient was readmitted to the hospital twice after the first operation, with symptoms of anastomotic stenosis. After diagnosed with *Candida*-induced gastrojejunostomosis stenosis by gastrointestinal endoscopy and endoscopic biopsy (**Figure 4**), fluconazole was indicated for 2 weeks. Blood sample withdrawn afterward showed no evidence of fungal agents and the anastomotic stenosis responded well to treatment (**Figure 4**). However, after 3 weeks, he came back with cachexia and symptoms of anastomotic stenosis. The gastrointestinal endoscopy showed no image of candidiasis but anastomotic stenosis due to a chronic inflammatory process. Therefore, we decided to perform a gastrojejunostomosis redo surgery, but the patient did not agree to do reoperation. Medical treatment was given, and symptoms improved.

## Discussion and conclusion

*Candida* species located on most of mucosal surfaces and mainly in the GIT, along with the skin and the mucosa in about 40% of healthy subjects (6, 7). Normally, the gastrointestinal fungi are well-controlled by beneficial gastrointestinal flora and gastric low pH (8). *Candida* species are also known as the most common human fungal pathogens, especially in patients with compromised immune systems (patients with HIV/AIDS, long-term steroid therapy, chemotherapeutic drugs, etc.) or in cachectic patients (6). In GIT, *Candida* infection, or candidiasis, is frequently seen in the esophagus, followed by the stomach, small intestine, and colon (9). Some studies have shown the correlation between *Candida* colonization and other diseases of GIT such as Crohn’s disease, ulcerative colitis, and gastric ulcers (9). Inflammation may also influence the GI flora, indirectly allowing *Candida* spp., with *C. albicans* particularly, to colonize (10). With the same mechanism, injudicious use of antibacterial agents could regulate the *Candida* spp. colonization (3). *Candida* species is the fourth most common cause of nosocomial sepsis and increase the rate of morbidity and mortality in critically ill patients, especially in patients undergoing extensive gastrointestinal surgery (11). Research by Kavyashree et al. divided risk factors of *Candida* infection or colonization into three groups (3):

- *Candida*-related factors: Infectious site and size, susceptibility of fungal stain.
- Patients’ factors: Use of prolonged antibacterial or antacid drugs, compromised immune systems, cachectic patients.
- Other factors: GIT devices, gastrointestinal surgery with mucosal injury.

All our two patients had a preoperative malnutritioned and debilitated condition, underwent extensive gastrointestinal surgery with multiple GIT’s reconstructions (one case was PD with extended lymphadenectomy and one case was total pancreatectomy with extended lymphadenectomy and segmental PV resection), as well as used of prolonged antibacterial (Cephalosporin third generation and Metronidazole for prophylaxis) and antacid drugs as well as total parenteral nutrition postoperatively. Otherwise, it could not be ruled out *Candida* infection intraoperatively due to weak intraoperative infectious control.

*Candida* organisms colonize in gastroduodenal ulcers, especially in large or perforated ones (12, 13). Fungal colonization inhibits healing process of GIT ulcer and inflammation in the mucosa layer. A study by Lee et al. reported that, among 62 cases of peritonitis due to peptic ulcer perforation, 23 cases were cultured positive for *Candida* spp. from peritoneal fluid, making up 37%. In another retrospective study, nine of the 22 cases (41%) found *Candida* in culture from intraoperative peritoneal fluid (14, 15). Both our patients had some risk factors: low preoperative body mass index (BMI), prolonged use of antibacterial or antacid drugs, and recent extensive gastrointestinal surgery. It is still unclear whether Candidiasis is the primary cause of ulceration, or the ulcer is secondarily infected during its progression. Most patients with gastric candidiasis occasionally have ulcer-like pain, nausea, vomiting, and weight loss, and, in some cases, ulcers could lead to gastrointestinal bleeding and perforative peritonitis. Ealy diagnosis can prevent some lethal complications and mortality. Invasive Candidiasis or nosocomial sepsis is a serious medical disorder characterized by fever, sepsis syndromes, respiratory distress, and, finally, multi−organ failure (16, 17). Nosocomial *Candida* sepsis was reported the fourth most common cause with high rate of morbidity and mortality (11). This highlights the importance of early diagnosis in suspicious cases with esophagogastroduodenoscopy (EGD) and endoscopic biopsy. A gold standard for diagnosis of gastric candidiasis is based on histopathological findings of fungal hyphae with microbiologically *Candida* species (3). The samples to isolate *Candida* varied according to clinical condition, and they could be in peritoneal fluid, suspected tissue samples through gastrointestinal endoscopic biopsy, and blood samples. Isolation of *Candida* in ascites or intraoperative tissue samples is more clinically significant than postoperative samples. Histological staining using either PAS or H&E stains demonstrates *Candida* elements like *Candida* spores and budding hyphae invading and destroying adjacent gastric wall, and surrounding regions of non-specific inflammation, with marked granulation tissue formation and inflammatory cells (eosinophils, macrophages, plasma cells, and lymphocytes) infiltration (3, 8). As we mentioned, *Candida* species located on most of mucosal surfaces and mainly in the GIT in about 40% of healthy subjects. Moreover, *Candida* species usually colonize in gastroduodenal mucosal injury such as ulcers, especially in large or perforated ones, so that gastrointestinal anastomosis could become a possible colonization of *Candida* species. We supposed that the overall condition of two patients (use of prolonged antibacterial or antacid drugs, compromised immune systems, cachectic patients, underwent gastrointestinal surgery with mucosal injury, and GIT devices) motivated the organism invasion of *Candida* species, leading to serious and systemic complications such as *Candida*-induced gastrojejunostomosis stenosis and perforation, visceral peritonitis, and nosocomial *Candida* sepsis.

In treatment, surgery is required for patients present with a visceral peritonitis following perforated fungal ulcer. In patients without severe complications that needs urgent interventions, invasive candidiasis required fluconazole. Antifungal regimen can also be applied in cases with recent abdominal surgery and recurrent gastrointestinal perforations or anastomotic leakages or in critically ill surgical patients at ICU and/or on prolonged ventilation and/or on parenteral nutrition (18). Fluconazole with a dose of 400 mg per day is the first choice, and caspofungin with a dose of 50 mg per day is recommended for fluconazole-resistant cases. The minimum period of antifungal agents is 2 weeks, until there is evidence of clearance of fungal agents in peritoneal fluid or blood samples (18). Cumulative length of hospital stay (CLOS) over 29 days was a strong predictor of fluconazole-resistant *Candida* spp (19). Surgical candidate was patient with candidiasis-induced anastomotic stenosis and perforation. Hence, both our patients required surgical interventions. In the first case, the patient was readmitted to the hospital with symptoms of perforative peritonitis, for which he underwent surgery and had *Candida* found in both gastrojejunostomosis ulcer and peritoneal fluid (**Figure 2**). In our second case, the patient was readmitted to the hospital twice after the first operation, with symptoms of anastomotic stenosis. After diagnosed with *Candida*-induced gastrojejunostomosis stenosis by gastrointestinal endoscopy and endoscopic biopsy (**Figure 4**), fluconazole was indicated for 2 weeks. Blood sample withdrawn afterward showed no evidence of fungal agents and the anastomotic stenosis responded well to treatment (**Figure 4**). However, after 3 weeks, he came back with cachexia and symptoms of anastomotic stenosis. EGD showed no image of fungal agents but anastomotic stenosis due to chronic inflammatory process. Therefore, we decided to perform a gastrojejunostomosis redo surgery, but the patient did not agree to do reoperation. Medical treatment was given, and symptoms improved.

## Conclusion

To the best of our knowledge, we present the first case report of gastrojejunostomosis candidiasis after PD in English literature. Clinical symptoms are non-specific but aggressive and present early postoperatively. Histological analysis are gold standards. Our report emphasizes the difficulty of diagnosis and management for this rare type of complication.

## Data availability statement

The original contributions presented in the study are included in the article/supplementary material. Further inquiries can be directed to the corresponding author.

## Ethics statement

Ethics approval of this study was given by the Research Ethics Committees of Bach Mai Hospital. Written informed consent was obtained from the participant for the publication of this case report.

## Author contributions

TN, HN and CN contributed equally as co-first authors, the main doctors to conceive the original idea and operate the patients; CN conceived and edited the manuscript; TL performed the operation and wrote the manuscript; LD edited the manuscript; VL and KD performed the operation; TT edited the manuscript. TN, HN, and the other authors discussed the results together and contributed to the final manuscript. All authors read and approved the final manuscript.

## Funding

This work was supported by number grant project: QGSP.2022.01. The funder has a responsibility of purchasing article processing charge (APC) and other related fees.

## Acknowledgments

The authors wish to thank to the Department of Gastrointestinal and Hepato-pancreato-biliary surgery, Bach Mai Hospital, Hanoi, Vietnam; the board of the Department of Gastroenterology and Hepatology, and University of Medicine and Pharmacy, Vietnam National University for their assistance during the time of in-hospital observation of our patients.

## Conflict of interest

The authors declare that the research was conducted in the absence of any commercial or financial relationships that could be construed as a potential conflict of interest.

## Publisher’s note

All claims expressed in this article are solely those of the authors and do not necessarily represent those of their affiliated organizations, or those of the publisher, the editors and the reviewers. Any product that may be evaluated in this article, or claim that may be made by its manufacturer, is not guaranteed or endorsed by the publisher.

## Abbreviations

BMI, body mass index; CLOS, cumulative length of hospital stay; CT, computed tomography; EGD, esophagogastroduodenoscopy; GIT, gastrointestinal tract; H&E, hematoxylin and eosin; ICU, intensive care unit; IPMN, intraductal papillary mucinous neoplasms; MCT, middle-chain triglyceride; PAS, periodic acid–Schiff; PD, pancreaticoduodenectomy; PDAC, pancreatic ductal adenocarcinoma; POD, postoperative day.
